# Fisetin ameliorates vascular smooth muscle cell calcification via DUSP1-dependent p38 MAPK inhibition

**DOI:** 10.18632/aging.206233

**Published:** 2025-04-02

**Authors:** Mehdi Razazian, Sheyda Bahiraii, Azmat Sohail, Markus Mandl, Isratul Jannat, Georg Beilhack, Ioana Alesutan, Jakob Voelkl

**Affiliations:** 1 Institute for Physiology and Pathophysiology, Johannes Kepler University Linz, Linz 4020, Austria; 2 Division of Nephrology and Dialysis, Department of Medicine III, Medical University of Vienna, Vienna 1090, Austria; 3 Department of Nephrology and Medical Intensive Care, Charité-Universitätsmedizin Berlin, Corporate Member of Freie Universität Berlin and Humboldt Universität zu Berlin, Berlin 13353, Germany; 4 DZHK (German Centre for Cardiovascular Research), Partner Site Berlin, Berlin 13347, Germany

**Keywords:** vascular calcification, vascular smooth muscle cells, fisetin, dual-specificity phosphatase 1, p38 MAPK

## Abstract

Medial vascular calcification is highly prevalent in advanced age and chronic kidney disease (CKD), where it is associated with increased risk for cardiovascular events and mortality. Vascular smooth muscle cells (VSMCs) actively regulate this process, which can be augmented by inflammation and cellular senescence. Thus, the present study investigated the impact of fisetin, a flavonol with anti-inflammatory and senolytic properties, on VSMC calcification. Fisetin treatment suppressed calcific marker expression and calcification of VSMCs as well as p38 MAPK phosphorylation induced by pro-calcific conditions. These effects were abolished by silencing of dual-specificity phosphatase 1 (DUSP1), a negative regulator of p38 MAPK activity. Moreover, knockdown of DUSP1 alone was sufficient to increase calcific marker expression in VSMCs, effects blunted by pharmacological p38 MAPK inhibition. Accordingly, DUSP1 knockdown aggravated calcification of VSMCs during pro-calcific conditions. In addition, fisetin ameliorated the effects of uremic conditions in VSMCs exposed to serum from dialysis patients. Fisetin also inhibited vascular calcification as well as calcific marker expression *ex vivo* in mouse aortic explants exposed to high phosphate and *in vivo* in a cholecalciferol overload mouse model. In conclusion, fisetin acts as a potent anti-calcific agent during VSMC calcification, an effect involving DUSP1-mediated regulation of p38 MAPK-dependent pro-calcific signaling.

## INTRODUCTION

Medial vascular calcification (VC) increases with advanced age [1]. This process is strongly accelerated by the uremic environment in chronic kidney disease (CKD) [2], which is considered a state of premature vascular aging [3]. VC increases vascular stiffness and elevates pulse pressure, resulting in impaired organ perfusion and increased left ventricular afterload [4]. Accordingly, the presence of calcifications in the arteries is associated with an increased mortality risk [5].

VC is the result of a complex process that ultimately culminates in deposition of calcium-phosphate in the vascular wall [6]. Under physiological conditions, extraosseous calcifications are actively prevented by calcification inhibitors, most importantly pyrophosphate, matrix GLA protein or fetuin-A [6]. In aged or diseased conditions, these mechanisms may be weakened and pro-calcific effects may be augmented in arterial tissue [2]. An especially important role in VC is attributed to vascular smooth muscle cells (VSMCs) [6]. These cells can take up pro-calcific functions and induce alteration of extracellular matrix, as well as release of pro-calcific vesicles and tissue-nonspecific alkaline phosphatase (ALPL), which cleaves the ubiquitous calcification inhibitor pyrophosphate [7]. The reprogramming of VSMCs towards a pro-calcific state is coordinated by various complex signaling pathways [8]. The mitogen-activated protein kinase (MAPK) p38 plays a prominent, but incompletely understood role in VC [9, 10]. P38 MAPK is activated by phosphorylation in response to various stimuli and inactivated by phosphatases such as dual-specificity phosphatase 1 (DUSP1, also known as MAPK phosphatase 1 or MKP1) [11]. P38 MAPK inhibition is sufficient to ameliorate VSMC calcification [12].

Phosphate has been recognized as a powerful stimulator of VSMC reprogramming and VC and may act, at least partly, through the formation of calciprotein particles [8]. Elevated phosphate conditions induce metabolic alterations with increased oxidative stress generation and activate inflammatory pathways to promote pro-calcific signaling in VSMCs [2, 13]. These alterations may involve VSMC senescence, inducing a proinflammatory senescence-associated secretory phenotype (SASP) [14]. Phosphate exposure is able to up-regulate senescence markers in VSMCs [15]. In turn, treatment of uremic rats with phosphate binders reduced expression of senescence markers in the vasculature [16]. Senolytic substances may eliminate senescent cells [17] and some have been shown to reduce VSMC calcification, such as quercetin [18], dasatinib [19], piperlongumine [20] or curcumin [21]. Thus, senolytics have been discussed as potential treatment options in uremic calcification, but their specificity and exact mechanisms might still be debatable.

One substance with senolytic potential is the flavonoid fisetin [22], which exhibits potent anti-inflammatory and antioxidant properties [23]. Fisetin increases lifespan in aged mice [24], attenuates renal fibrosis in cisplatin nephrotoxicity [25] and ameliorates atherosclerosis [26]. This study, therefore, investigated the vasculo-protective potential of fisetin in phosphate-induced VSMC calcification and the underlying mechanisms.

## RESULTS

**Figure 1:**
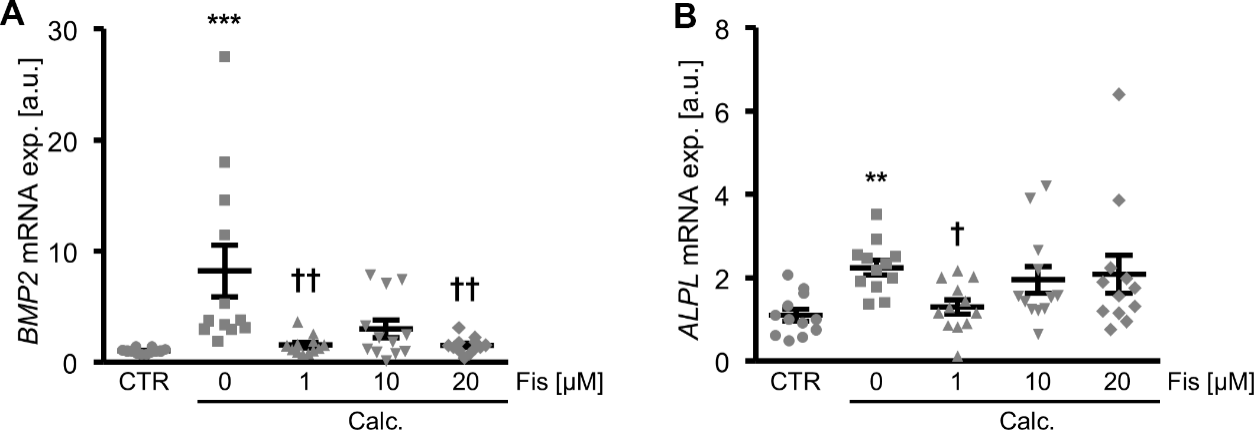
**Dose-dependent effects of fisetin on calcific marker expression in VSMCs during pro-calcific conditions.** Relative mRNA expression (n=12) of *BMP2* (**A**) and *ALPL* (**B**) in HAoSMCs treated for 48h with control (CTR) or calcification medium (Calc.) without and with the indicated concentrations of fisetin (Fis, 0 - 20 μM). **(p<0.01), ***(p<0.001) significant vs. control group; †(p<0.05), ††(p<0.01) significant vs. Calc.-treated group.

To investigate a possible impact of fisetin on VSMC calcification, a first series of experiments was performed in cultured primary human aortic VSMCs during control or pro-calcific conditions with addition of calcium and the phosphate donor β-glycerophosphate, in the absence and presence of increasing concentrations of fisetin (0 – 20 μM). As illustrated in Figure 1, calcification medium significantly up-regulated *BMP2* and *ALPL* mRNA expression in VSMCs, effects significantly suppressed in the presence of 1 μM fisetin concentration. Lower fisetin concentrations did not significantly affect calcification medium-induced *BMP2* and *ALPL* mRNA expression (Supplementary Figure 1).

**Figure 2:**
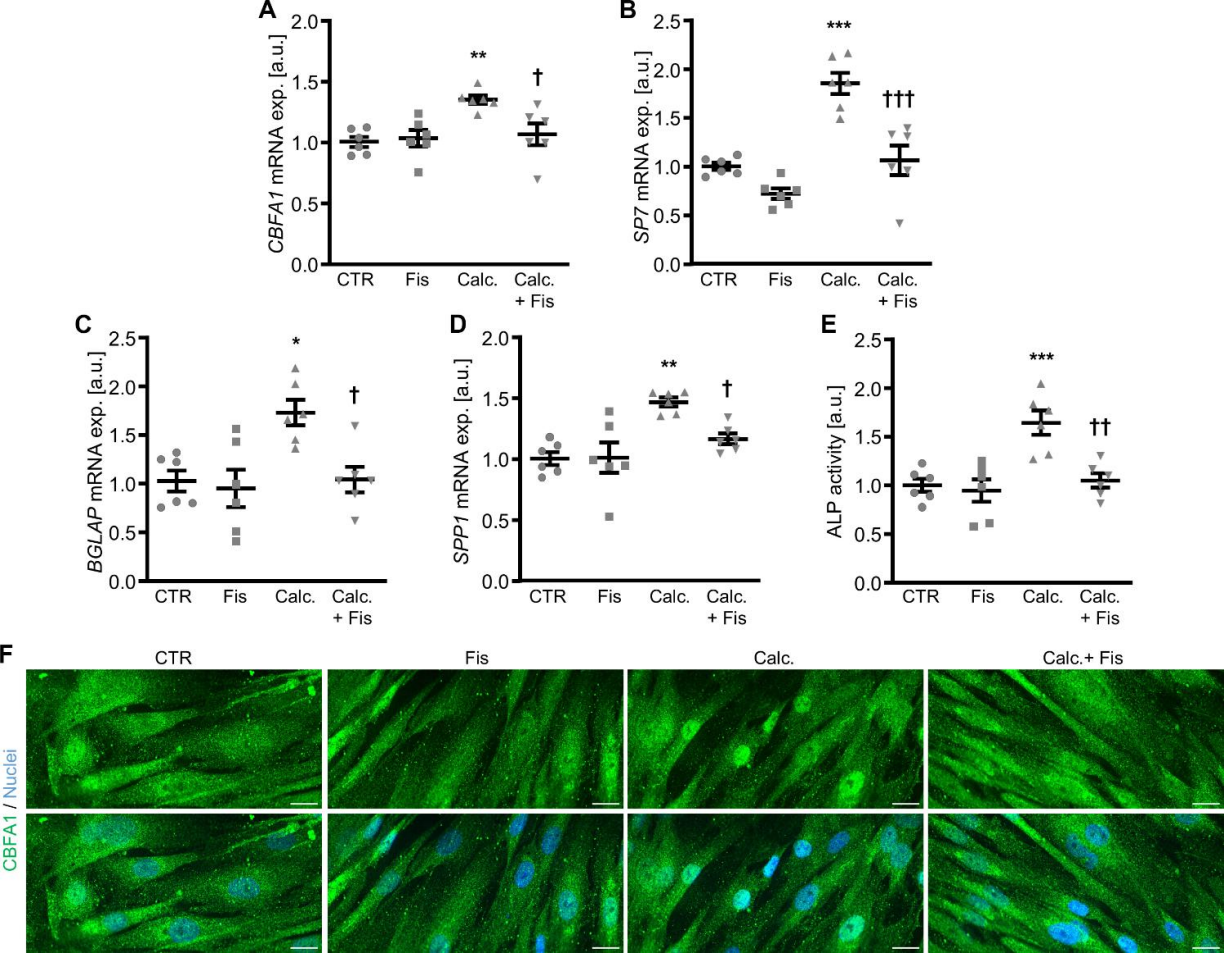
**Effects of fisetin on pro-calcific signaling in VSMCs during pro-calcific conditions.** Relative mRNA expression (n=6) of *CBFA1* (**A**), *SP7* (**B**), *BGLAP* (**C**) and *SPP1* (**D**) in HAoSMCs treated for 48h with control (CTR) or calcification medium (Calc.) without and with 1 μM fisetin (Fis). (**E**) Normalized ALP activity (n=6) in HAoSMCs treated for 7d with control (CTR) or calcification medium (Calc.) without and with 1 μM fisetin (Fis). *(p<0.05), **(p<0.01), ***(p<0.001) significant vs. control group; †(p<0.05), ††(p<0.01), †††(p<0.001) significant vs. Calc.-treated group. (**F**) CBFA1 (green) and nuclei (blue) shown by confocal imaging in HAoSMCs treated for 48h with control (CTR) or calcification medium (Calc.) without and with 1 μM fisetin (Fis). Scale bar: 20 μm.

**Figure 3:**
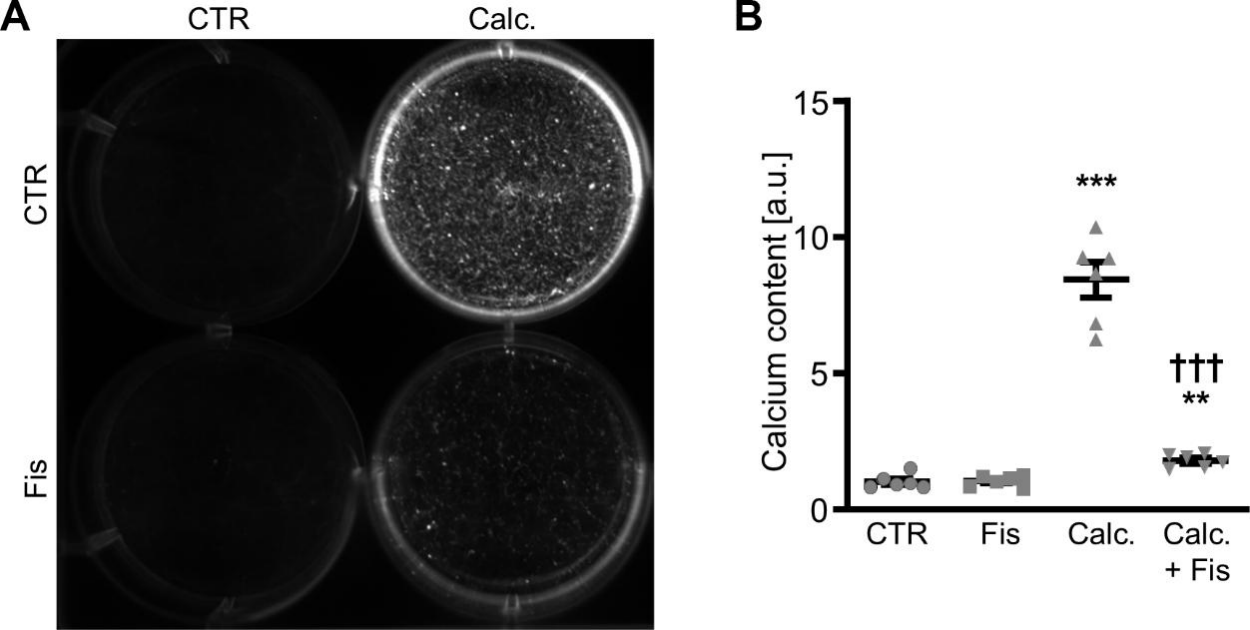
**Effects of fisetin on calcification of VSMCs during pro-calcific conditions.** Calcification detected by Osteosense fluorescence imaging (**A**) and normalized calcium content (n=6, **B**) in HAoSMCs treated for 11d with control (CTR) or calcification medium (Calc.) without and with 1 μM fisetin (Fis). Calcified areas: white pseudocolor. **(p<0.01), ***(p<0.001) significant vs. control group; †††(p<0.001) significant vs. Calc.-treated group.

The calcification medium-induced increase of pro-calcific markers *CBFA1*, *SP7*, *BGLAP* and *SPP1* mRNA expression (Figure 2A–2D), ALP activity (Figure 2E) as well as CBFA1 nuclear localization (Figure 2F) were all suppressed by 1 μM fisetin supplementation. Co-treatment with fisetin reduced expression of markers associated with senescence of VSMCs promoted by calcification medium, as shown by the mRNA expression of *CDKN1A* and *GLB1* and further indicated by senescence-associated (SA)-β-galactosidase staining (Supplementary Figure 2). Treatment with fisetin alone did not consistently modify pro-calcific and senescence markers expression at 1 μM concentration, but high concentrations of fisetin (20 μM) had adverse effects in VSMCs (Supplementary Figure 3). Moreover, pre-treatment of VSMCs with 1 μM fisetin did not significantly modify calcification medium-induced pro-calcific marker expression (Supplementary Figure 4). More importantly, as illustrated by Osteosense fluorescence imaging and determination of calcium content (Figure 3), co-treatment with 1 μM fisetin significantly reduced calcification of VSMCs exposed to calcification medium. Taken together, fisetin inhibited pro-calcific signaling and calcification of VSMCs induced by mineral stress during high phosphate and calcium exposure.

**Figure 4:**
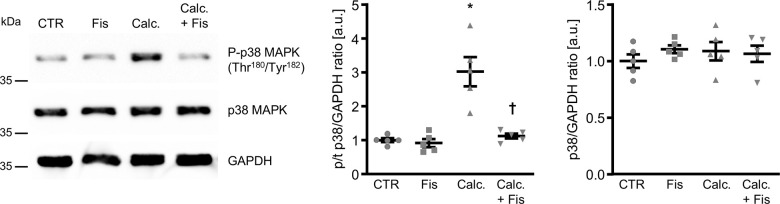
**Effects of fisetin on p38 MAPK phosphorylation in VSMCs during pro-calcific conditions.** Representative Western blots and normalized phospho-p38 and total p38 MAPK protein abundance (n=5) in HAoSMCs treated for 30 min with control (CTR) or calcification medium (Calc.) without and with 1 μM fisetin (Fis). *(p<0.05) significant vs. control group; †(p<0.05) significant vs. Calc.-treated group.

**Figure 5:**
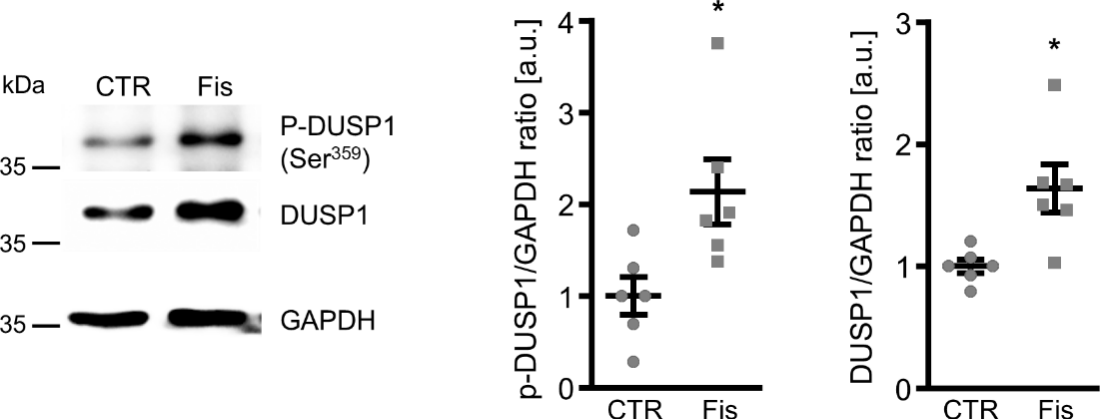
**Effects of fisetin on dual-specificity phosphatase 1 regulation in VSMCs.** Representative Western blots and normalized phospho-DUSP1 and total DUSP1 protein abundance (n=6) in HAoSMCs treated for 30 min with control (CTR) or 1 μM fisetin (Fis). *(p<0.05) significant vs. control group.

To elucidate the underlying mechanisms of the anti-calcific effects of fisetin in VSMCs, its potential to interfere with p38 MAPK activation was explored. As illustrated in Figure 4, calcification medium induced phosphorylation of p38 MAPK in VSMCs, effects significantly blunted in the presence of fisetin. Subsequently, the role of dual-specificity phosphatase 1 (DUSP1), a negative regulator of p38 MAPK activity was investigated. As shown in Figure 5, fisetin increased total DUSP1 protein abundance in VSMCs as well as the phosphorylation of DUSP1 at Ser^359^, a direct phosphorylation site for p44/42 MAPK that could inhibit DUSP1 degradation via the ubiquitination pathway [27]. Moreover, fisetin increased the abundance of phosphorylated and total p44/42 MAPK protein in VSMCs (Supplementary Figure 5). Pharmacological inhibition of p44/42 MAPK with LY3214996 blunted fisetin-induced phosphorylation of DUSP1 at Ser^359^ (Supplementary Figure 6).

**Figure 6:**
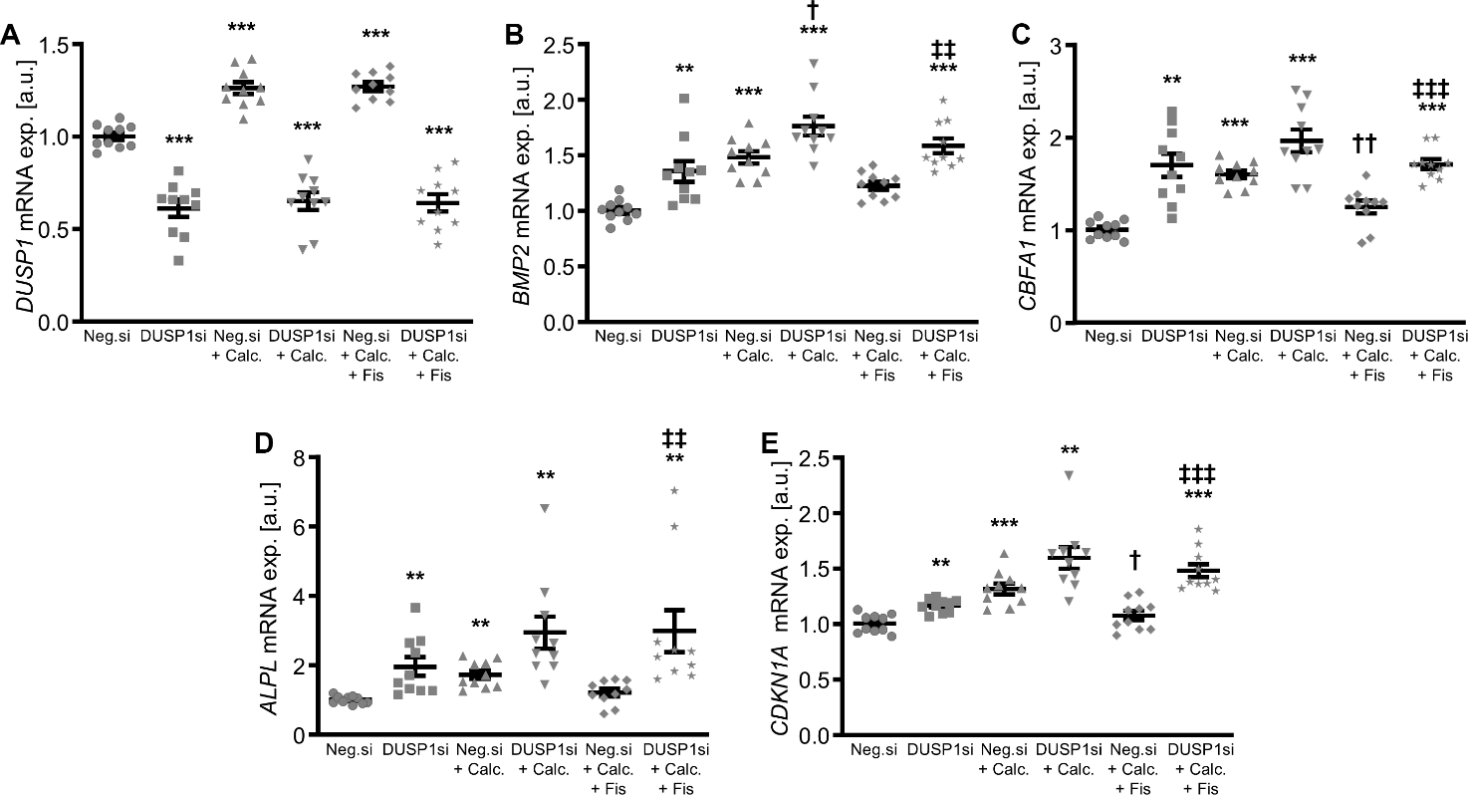
**Effects of dual-specificity phosphatase 1 knockdown on anti-calcifying properties of fisetin in VSMCs.** Relative mRNA expression (n=10) of *DUSP1* (**A**), *BMP2* (**B**), *CBFA1* (**C**), *ALPL* (**D**) and *CDKN1A* (**E**) in HAoSMCs transfected for 72h with negative control (Neg.si) or DUSP1 (DUSP1si) siRNA and treated for 48h with control (CTR) or calcification medium (Calc.) without and with 1 μM fisetin (Fis). **(p<0.01), ***(p<0.001) significant vs. Neg.si group; †(p<0.05), ††(p<0.01) significant vs. Neg.si+Calc.-treated group; ‡‡(p<0.01), ‡‡‡(p<0.001) significant between Neg.si+Calc.+Fis- and DUSP1si+Calc.+Fis-treated groups.

**Figure 7:**
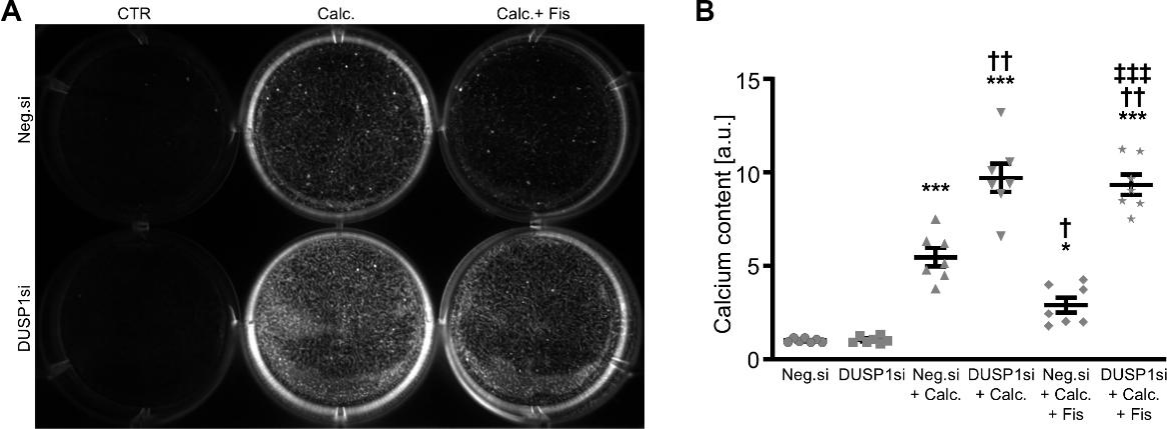
**Effects of dual-specificity phosphatase 1 knockdown on the protective role of fisetin during VSMC calcification.** Calcification detected by Osteosense fluorescence imaging (**A**) and normalized calcium content (n=7, **B**) in HAoSMCs transfected with negative control (Neg.si) or DUSP1 (DUSP1si) siRNA and treated for 11d with control (CTR) or calcification medium (Calc.) without and with 1 μM fisetin (Fis). Calcified areas: white pseudocolor. *(p<0.05), ***(p<0.001) significant vs. Neg.si group; †(p<0.05), ††(p<0.01) significant vs. Neg.si+Calc.-treated group; ‡‡‡(p<0.001) significant between Neg.si+Calc.+Fis- and DUSP1si+Calc.+Fis-treated groups.

In further experiments, the endogenous expression in VSMCs was suppressed by silencing of the *DUSP1* gene using small interfering RNA (siRNA), during control and pro-calcific conditions with and without fisetin supplementation. As a result, transfection with DUSP1 siRNA significantly reduced *DUSP1* expression in VSMCs as compared to negative control siRNA-transfected cells (Figure 6A). Calcification medium increased *DUSP1* mRNA expression in VSMCs (Figure 6A and Supplementary Figure 7), which was not significantly affected by fisetin (Figure 6A). Knockdown of DUSP1 alone was sufficient to significantly up-regulate mRNA expression of pro-calcific markers in VSMCs (Figure 6B–6E). Furthermore, DUSP1 silencing abolished the protective effects of fisetin during pro-calcific conditions. In accordance, silencing of DUSP1 aggravated VSMC calcification induced by calcification medium and virtually abrogated the anti-calcific properties of fisetin (Figure 7).

**Figure 8:**
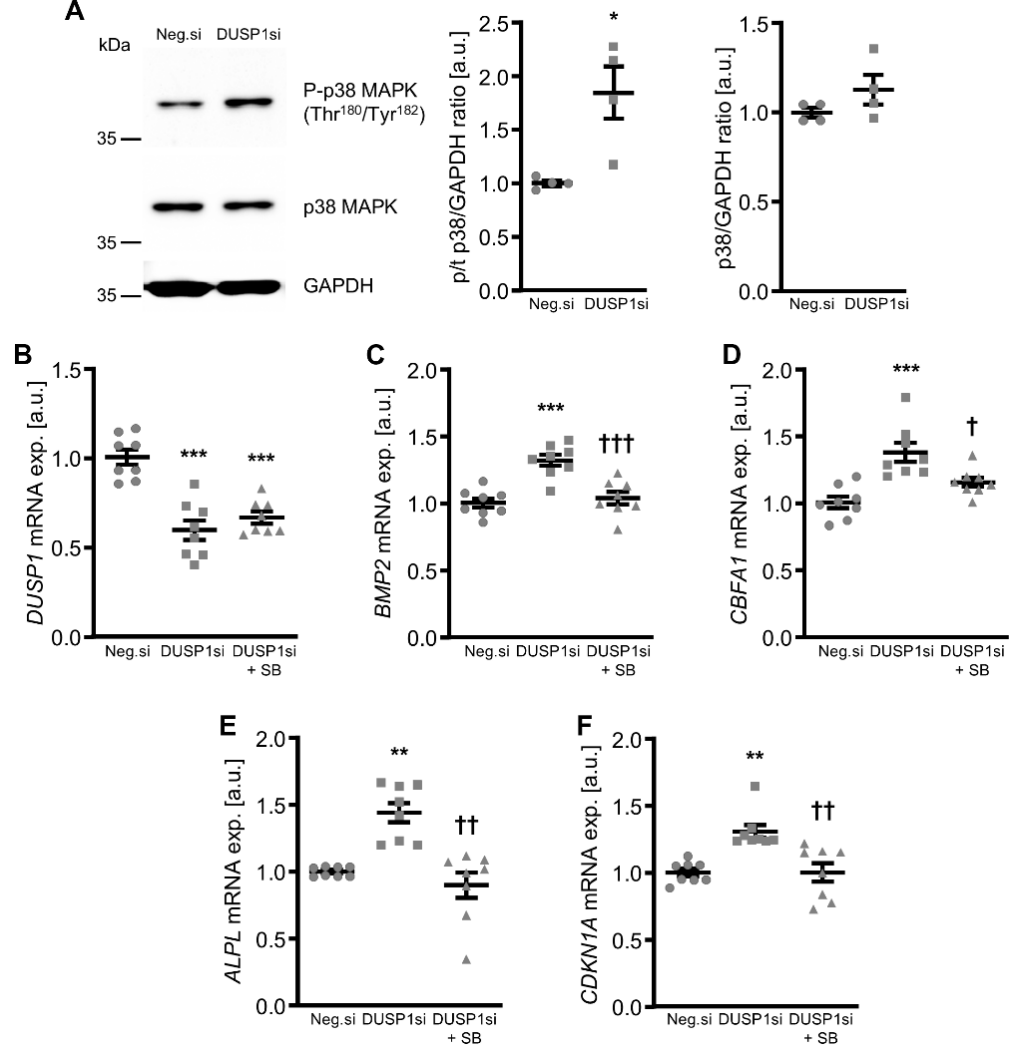
**Role of p38 MAPK in dual-specificity phosphatase 1 knockdown-induced calcific marker expression in VSMCs.** Representative Western blots and normalized phospho-p38 and total p38 MAPK protein abundance (n=4, **A**) in HAoSMCs transfected for 24h with negative control (Neg.si) or DUSP1 (DUSP1si) siRNA. Relative mRNA expression (n=8) of *DUSP1* (**B**), *BMP2* (**C**), *CBFA1* (**D**), *ALPL* (**E**) and *CDKN1A* (**F**) in HAoSMCs transfected for 72h with negative control (Neg.si) or DUSP1 (DUSP1si) siRNA and treated without and with 10 μM p38 MAPK inhibitor SB203580 (SB). *(p<0.05), **(p<0.01), ***(p<0.001) significant vs. Neg.si group; †(p<0.05), ††(p<0.01), †††(p<0.001) significant vs. DUSP1si group.

Moreover, knockdown of DUSP1 was sufficient to increase phosphorylation of p38 MAPK in VSMCs (Figure 8A), but DUSP1 knockdown did not significantly affect SAPK/JNK or p44/42 MAPK phosphorylation (Supplementary Figure 8). Pharmacological inhibition of p38 MAPK with SB203580 significantly blunted the increased *BMP2*, *CBFA1*, *ALPL* and *CDKN1A* mRNA expression in DUSP1-silenced VSMCs (Figure 8C–8F). Treatment with the SB203580 inhibitor alone did not significantly modify *DUSP1* or calcific marker mRNA expression in VSMCs (Supplementary Figure 9). Thus, knockdown of DUSP1 increased p38 MAPK-dependent pro-calcific signaling and aggravated calcification of VSMCs.

**Figure 9:**
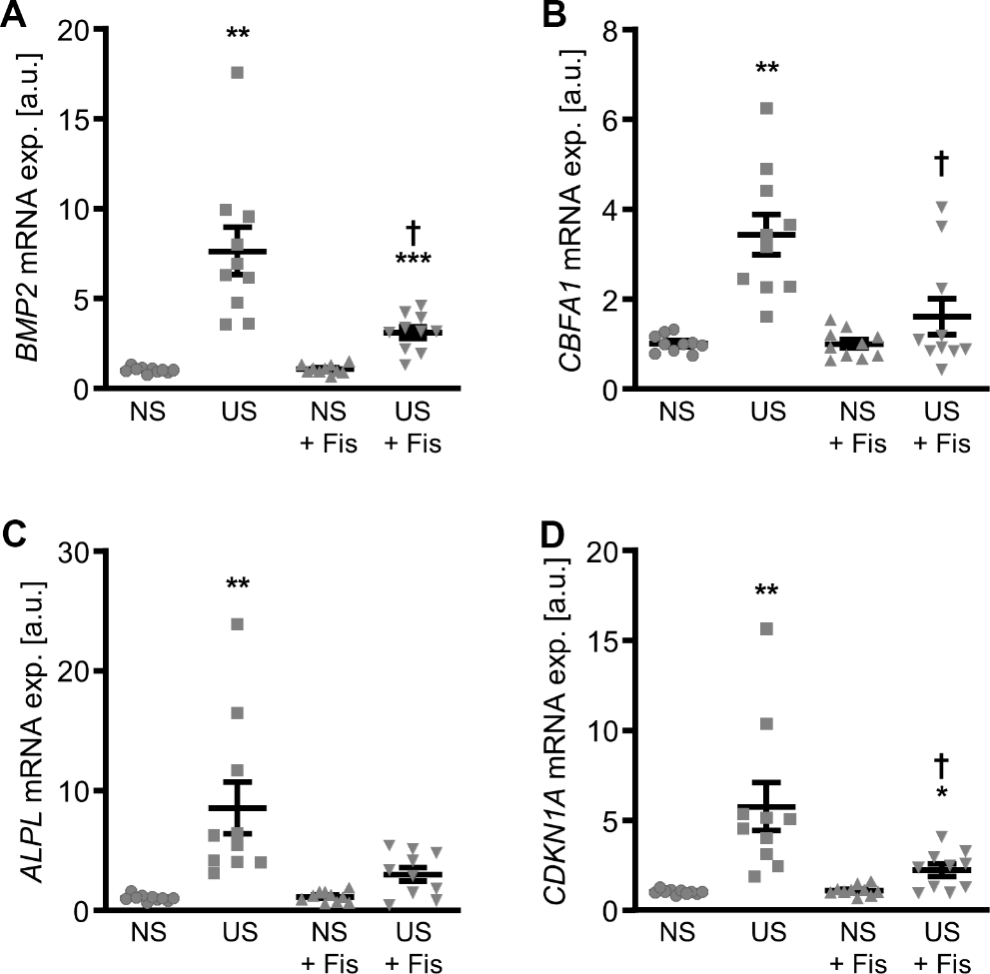
**Effects of fisetin on calcific marker expression in VSMCs during uremic conditions.** Relative mRNA expression (n=10) of *BMP2* (**A**), *CBFA1* (**B**), *ALPL* (**C**) and *CDKN1A* (**D**) in HAoSMCs treated for 24h with 15% normal serum (NS) or uremic serum (US) without and with 1 μM fisetin (Fis). *(p<0.05), **(p<0.01), ***(p<0.001) significant vs. NS group; †(p<0.05) significant vs. respective serum alone group.

Next experiments explored the effects of fisetin on pro-calcific marker expression in VSMCs during uremic conditions. Exposure of VSMCs to uremic serum from hemodialysis patients significantly increased *BMP2*, *CBFA1*, *ALPL* and *CDKN1A* mRNA expression as compared to VSMCs exposed to control serum from healthy volunteers (Figure 9). Treatment with fisetin significantly suppressed uremic serum-induced *BMP2*, *CBFA1* and *CDKN1A* mRNA expression and tended to reduce *ALPL* mRNA expression, a difference, however, not reaching statistical significance (p=0.0551). Thus, fisetin treatment induced protective effects in VSMCs during uremic conditions.

Additional experiments explored the effects of fisetin following angiotensin II stimulation. Again, fisetin ameliorated the expression of pro-calcific markers in VSMCs during angiotensin II treatment (Supplementary Figure 10).

**Figure 10:**
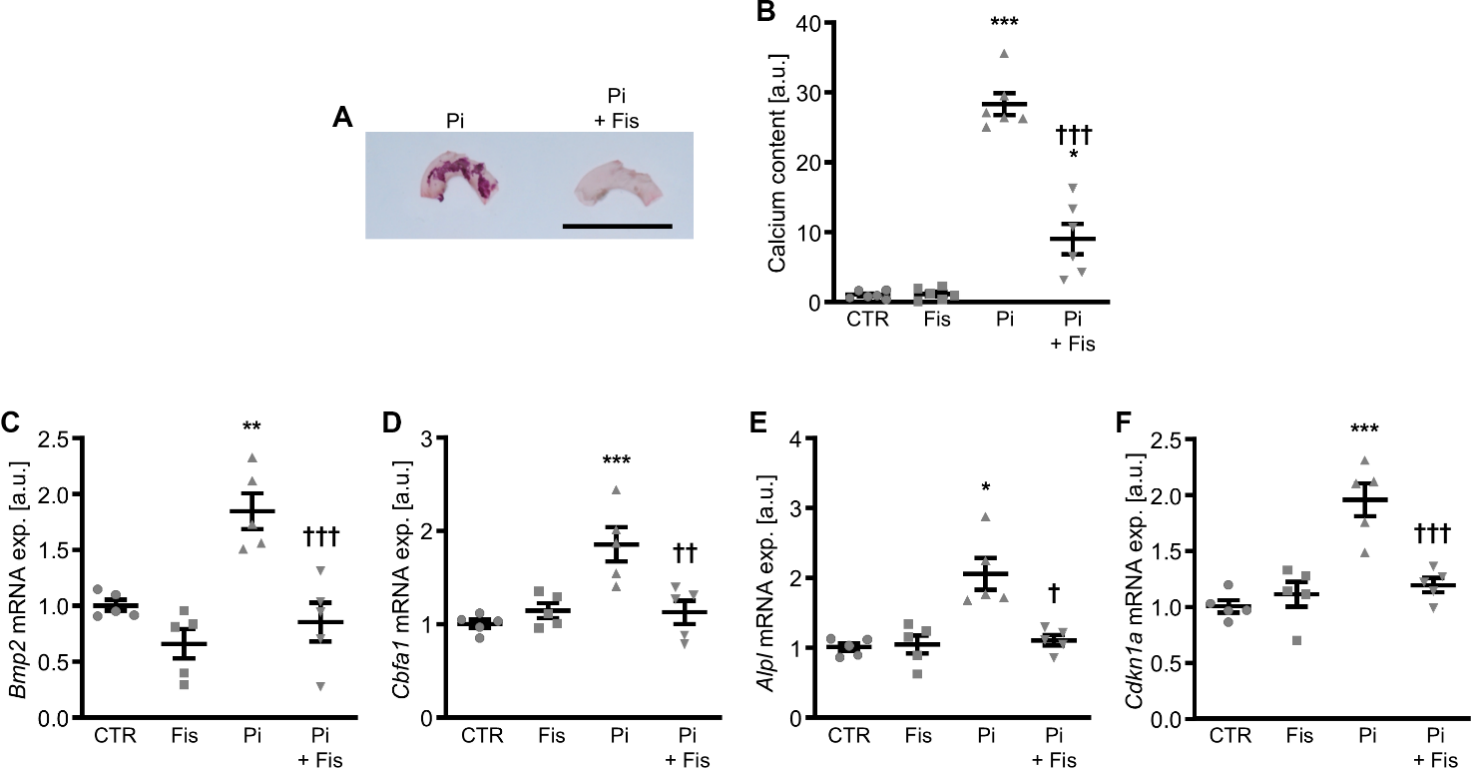
**Effects of fisetin *ex vivo* in calcifying mouse aortic explants.** Alizarin Red staining (**A**) of mouse aortic arches cultured for 7d in medium supplemented with 1.6 mM phosphate (Pi) without and with 1 μM fisetin (Fis). Calcification: red staining; scale bar: 5 mm. Normalized calcium content (n=6, **B**) in mouse aortic explants cultured for 7d in medium supplemented with control (CTR) or 1.6 mM phosphate (Pi) without and with 1 μM fisetin (Fis). Relative mRNA expression (n=5) of *Bmp2* (**C**), *Cbfa1* (**D**), *Alpl* (**E**) and *Cdkn1a* (**F**) in mouse aortic explants cultured for 7d in medium supplemented with control (CTR) or 1.6 mM phosphate (Pi) without and with 1 μM fisetin (Fis). *(p<0.05), **(p<0.01), ***(p<0.001) significant vs. control group; †(p<0.05), ††(p<0.01), †††(p<0.001) significant vs. Pi-treated group.

To confirm the anti-calcific properties of fisetin, additional experiments were performed *ex vivo* in mouse aortic explants cultured during control or high phosphate conditions without and with co-treatment with fisetin. As shown in Figure 10, fisetin reduced calcification as well as the increased mRNA expression of *Bmp2*, *Cbfa1*, *Alpl* and *Cdkn1a* induced by phosphate exposure in mouse aortic explants. In addition, fisetin significantly reduced the abundance of phosphorylated p38 Mapk and tended to reduce total p38 Mapk protein abundance (p=0.0798) in mouse aortic explants during high phosphate conditions (Supplementary Figure 11).

**Figure 11:**
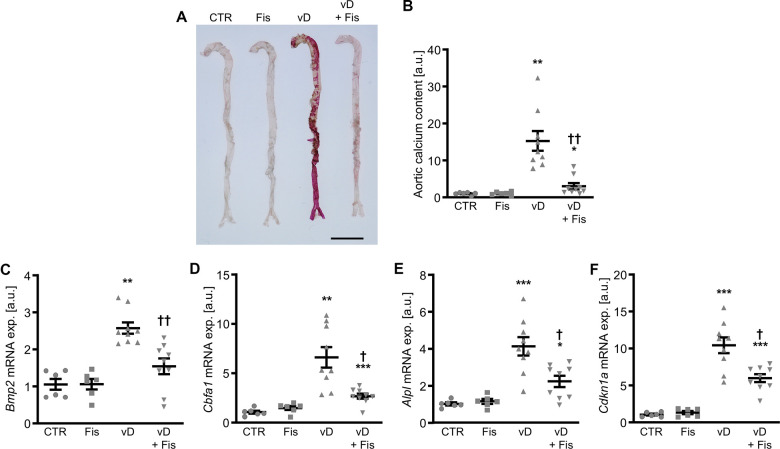
**Effects of fisetin *in vivo* during cholecalciferol overload.** Alizarin Red staining (**A**) of aortas from mice receiving vehicle (CTR) or high-dosed cholecalciferol (vD) without and with fisetin (Fis). Calcification: red staining; scale bar: 5 mm. Normalized calcium content (n=6-9, **B**) in aortic tissue from mice receiving vehicle (CTR) or high-dosed cholecalciferol (vD) without and with fisetin (Fis). Relative mRNA expression (n=6-9) of *Bmp2* (**C**), *Cbfa1* (**D**), *Alpl* (**E**) and *Cdkn1a* (**F**) in aortic tissue from mice receiving vehicle (CTR) or high-dosed cholecalciferol (vD) without and with fisetin (Fis). *(p<0.05), **(p<0.01), ***(p<0.001) significant vs. control group; †(p<0.05), ††(p<0.01) significant vs. vD-treated group.

Further experiments investigated the effects of fisetin *in vivo* in the cholecalciferol-induced VC mouse model. As shown by alizarin Red staining and quantification of calcium content, cholecalciferol overload induced aortic calcification in mice, effects significantly reduced by additional fisetin treatment (Figure 11A, 11B). Furthermore, fisetin suppressed cholecalciferol-induced *Bmp2*, *Cbfa1*, *Alpl* and *Cdkn1a* mRNA expression in the aortic tissues (Figure 11C–11F). High-dosed cholecalciferol significantly increased serum calcium and altered cystatin C, phosphate and fetuin A concentrations, while fisetin ameliorated most effects of cholecalciferol treatment (Supplementary Table 1).

## DISCUSSION

**Figure 12:**
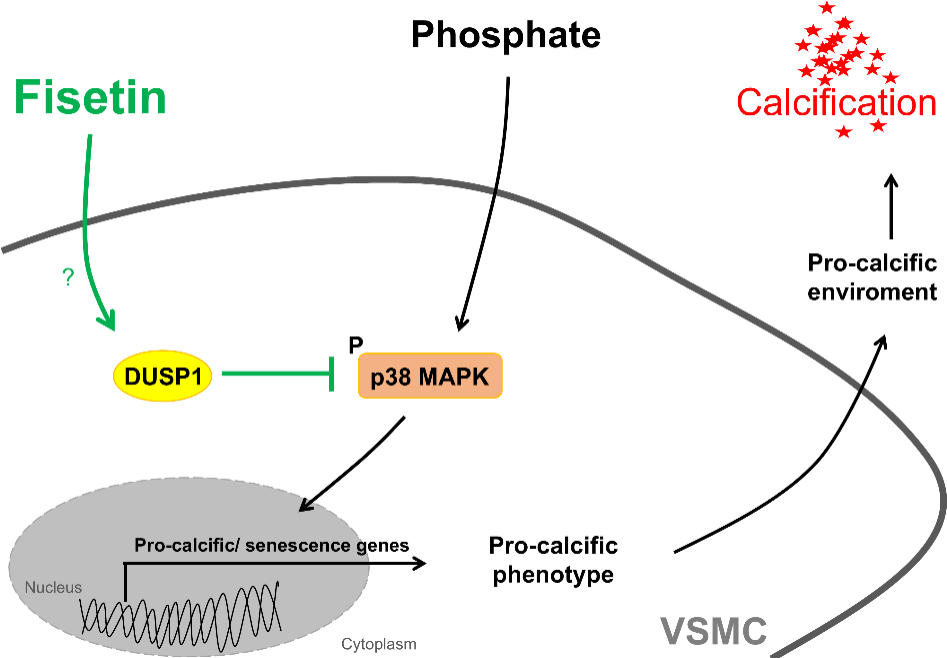
**Schematic illustration of mechanisms involved in the protective effects of fisetin during VSMC calcification.** Mineral stress with disturbed phosphate and calcium homeostasis may trigger activation of pro-calcific signaling including phosphorylation and activation of p38 MAPK in VSMCs, which leads to a pro-calcific environment causing vascular calcification. Fisetin, a natural flavonol, induces the dual-specificity phosphatase 1 (DUSP1). Fisetin thereby inactivates p38 MAPK signaling through DUSP1 and inhibits further pro-calcific signaling and calcification of VSMCs.

This study discloses a powerful anti-calcific effect of fisetin in VSMCs during mineral stress, a condition typically observed in CKD [2]. Mechanistically, fisetin requires the phosphatase DUSP1 to inhibit p38 MAPK in order to mediate its protective effect on VSMC calcification (Figure 12).

Fisetin has previously been associated with protective effects in the vasculature and prevents neointimal hyperplasia [28], atherosclerosis [26], glucose-induced inflammation [29] and endothelial dysfunction [30] in animal models or cell culture. Especially the ability of fisetin to decrease vascular senescence has been well established [30, 31]. Also in the kidney, fisetin is associated with attenuation of fibrosis [25, 32]. The current observations now extend the protective effects of fisetin on the calcifying vasculature *in vitro*, *ex vivo* and *in vivo*. Although fisetin treatment altered calcium-phosphate homeostasis in cholecalciferol-treated mice and this might affect the calcification response, a direct effect on the vasculature is supported by aortic explant calcification model and cell culture experiments. A potential translational relevance of these observations for the CKD environment is underscored by the protective effects of fisetin in VSMCs exposed to uremic serum.

Mechanistically, the effects of fisetin appear to be mediated by inhibition of p38 MAPK during pro-calcific conditions. P38 MAPK appears to have a central role in pro-calcific signaling of VSMCs [9, 10, 33]. P38 MAPK inhibition has been shown to ameliorate VSMC calcification after phosphate exposure, while ERK1/2 MAPK or SAPK/JNK inhibition showed no effect [12]. The downstream effects of p38 MAPK activation in VC apparently are mediated through a mechanism involving RUNX2/CBFA1 [10]. P38 MAPK also exerts a critical role in the intracellular signaling pathways of inflammatory mediators, which are able to augment VC.

Accordingly, the pro-calcific effects of CRP [33], IL18 [34], ceramide [9] or angiotensin II [35] involve p38 MAPK.

P38 kinases react to a variety of cellular stress conditions and control a diverse array of cellular functions [11]. Phosphatases such as DUSP1 are induced by p38 MAPK and limit or terminate p38 MAPK signaling [11]. The current observations identify DUSP1 as a novel regulator of VSMC calcification. DUSP1 is expressed in VSMCs and plays an important role in maintaining the contractile phenotype by regulating p38 MAPK [36]. Fisetin reduces p38 MAPK phosphorylation in calcifying VSMCs apparently through DUSP1. Similarly, fisetin inhibits ubiquitination and proteasomal degradation of DUSP1 in osteoclasts and thereby limits p38 MAPK signaling [37]. In T-cells, angiotensin II increases proteasome activity and degrades DUSP1 [38]. Angiotensin II and aldosterone attenuate DUSP1 expression in VSMCs [30]. In accordance, we observed a reduced pro-calcific effect of angiotensin II in VSMCs treated with fisetin. Mechanistically, fisetin might activate ERK1/2 MAPK [39]. ERK1/2 could phosphorylate DUSP1 on Ser^359^, leading to its stabilization [27], but other ERK1/2-dependent phosphorylation sites could actually promote DUSP1 proteasomal degradation [40]. Therefore, the regulatory network of DUSP1 activation and its effect on MAPK appear intricate and multi-factorial [41]. The exact mechanisms how fisetin regulates DUSP1 are currently unknown.

Although the gene silencing experiments indicate a major role for DUSP1 in the anti-calcific effects of fisetin, the current experiments cannot rule out involvement of other pathways or mechanisms. Also, ubiquitination of NRF2 is negatively regulated by fisetin [42]. NRF2 is a powerful modulator of VC [43]. Other pathways might therefore contribute to the effects of fisetin in phosphate-treated VSMCs. In addition, the role of cellular senescence in the current observations should be interpreted with caution. P38 MAPK is important for the development of a senescence-associated secretory phenotype (SASP) [44] and the increased expression of senescence markers in calcifying VSMCs are ameliorated by fisetin. However, the role of DUSP1 in senescence appears complex [45, 46] and the primary effect of fisetin in this model may be mediated through other mechanisms than directly through senolytic properties [22]. In neuronal cells, DUSP1 is polyubiquitylated and increasingly degraded in response to glutamate-induced oxidative stress [47]. Thus, oxidative stress might shift the balance of DUSP1 expression and degradation. Increased oxidative stress is also a hallmark of phosphate-induced VC [2]. In turn, fisetin has been attributed with substantial antioxidant properties [23], which could therefore be involved in its protective effect during VC and subsequently alter expression of senescence markers.

Fisetin treatment has already been used in animal models of CKD and renal fibrosis [25, 32]. In addition, first clinical studies are being conducted on the beneficial effects of fisetin [48]. While the reduced renal function and uremic environment in CKD patients warrants caution, our observations suggest a potential benefit of fisetin treatment on VC. However, the current observations are limited by the model systems used. The artificial cell culture environment does not allow extrapolation to the *in vivo* situation, especially in terms of effective concentrations of fisetin. The cholecalciferol model rapidly induces VC, but differs from the situation in CKD, where active vitamin D levels are typically reduced. Therefore, CKD-specific or sex-dependent effects cannot be interpreted and further translational studies are required to delineate whether the putative benefits outweigh the risks of fisetin treatment in CKD.

In conclusion, this study shows a novel role of fisetin as powerful protective agent during phosphate-induced VSMC calcification. Mechanistically, this effect identifies a critical role of DUSP1 in the pro-calcific p38 MAPK signaling of VSMCs during calcifying conditions.

## MATERIALS AND METHODS

### Cell culture

Primary human aortic smooth muscle cells (HAoSMCs, Fisher Scientific and Cell Applications) were routinely cultured as described previously [49–51] and used in experiments up to passage 12. HAoSMCs were treated for the indicated times with 10 mM β-glycerophosphate and 1.5 mM CaCl_2_ (Sigma Aldrich) as calcification medium [52], 1 μM or the indicated concentrations of fisetin (stock in DMSO, HY-N0182, MedChemExpress), 10 μM of p38 MAPK inhibitor SB203580 (stock in DMSO, 13067, Cayman Chemical) [33] and 1 μM of p44/42 MAPK inhibitor LY3214996 (stock in DMSO, HY-101494, MedChemExpress) [53]. Where indicated, cells were pre-treated for 48 hours with 1 μM fisetin prior to treatments. After informed consent, serum was collected from dialysis patients (uremic serum, US) or control serum from apparently healthy individuals with absence of known CKD (normal serum, NS) and stored at -80° C. HAoSMCs were treated with 15% uremic serum or control serum [52]. Treatments with control (age 53.6 ± 1.7 years) or uremic (age 63.3 ± 3.0 years) serum was sex-matched (n =5 female / n=5 male). Where indicated, HAoSMCs were transfected with 10 nM DUSP1 (ID: s4363) or negative control (ID: 4390843) siRNA using siPORT amine transfection reagent (all from Fisher Scientific). Treatment with equal amounts of vehicle was used as control. For long-term treatments, fresh medium with agents were added every 2-3 days.

### Animal experiments

Calcification was induced in female C57BL/6 mice [54] by daily injections with cholecalciferol of 500 IU/g on d1-d3. Mice were gavaged daily with control or 100 mg/kg fisetin (5% ETOH, 55% PEG 300, 40% Water). Blood was collected by retroorbital puncture on d6 and tissues were snap frozen or stained with Alizarin Red (0.0016% in 0.5% KOH) [55]. Serum concentrations of calcium and phosphate were determined by using a photometric method (FUJI Dri-Chem Nx700). Serum concentrations of Cystatin C (RD291009200R, BioVendor) and Fetuin A (MFTA00, R&D Systems) were determined by ELISA.

### *Ex vivo* mouse aortic explants culture

C57BL/6 mice were sacrificed by cervical dislocation in isoflurane anaesthesia and aortic tissues were rapidly excised, cut into rings and cultured for 1 hour or 7 days in DMEM high glucose medium supplemented with 5% FBS, 100 U/ml penicillin and 100 μg/ml streptomycin and 0.25 μg/ml Fungizone (all from Fisher Scientific). Aortic rings were treated with 1.6 mM sodium phosphate buffer (Sigma Aldrich) and 1 μM fisetin (stock in DMSO, HY-N0182, MedChemExpress). For long-term treatments, fresh medium with agents were added every 2 days. Tissues were snap frozen or stained with Alizarin Red (0.0016% in 0.5% KOH).

### RNA isolation and RT-PCR

Total RNA was isolated by using Trizol Reagent and reverse transcription was performed by using oligo(dT)_12-18_ primers and SuperScript III Reverse Transcriptase (all from Fisher Scientific). RT-PCR was performed with iQ Sybr Green Supermix (Bio-Rad Laboratories) and the primers listed below (Fisher Scientific) [56–58]. Relative mRNA fold changes were calculated by the 2^-ΔΔCt^ method using GAPDH as housekeeping gene.

Human primers:

*ALPL* fw: GGGACTGGTACTCAGACAACG; *ALPL* rev: GTAGGCGATGTCCTTACAGCC; *BGLAP* fw: CACTCCTCGCCCTATTGGC; *BGLAP* rev: CCCTCCTGCTTGGACACAAAG; *BMP2* fw: TTCGGCCTGAAACAGAGACC; *BMP2* rev: CCTGAGTGCCTGCGATACAG; *CBFA1* fw: GCCTTCCACTCTCAGTAAGAAGA; *CBFA1* rev: GCCTGGGGTCTGAAAAAGGG; *CDKN1A* fw: TGTCCGTCAGAACCCATGC; *CDKN1A* rev: AAAGTCGAAGTTCCATCGCTC; *DUSP1* fw: AGTACCCCACTCTACGATCAGG; *DUSP1* rev: GAAGCGTGATACGCACTGC; *GAPDH* fw: GAGTCAACGGATTTGGTCGT; *GAPDH* rev: GACAAGCTTCCCGTTCTCAG; *GLB1* fw: TATACTGGCTGGCTAGATCACTG; *GLB1* rev: GGCAAAATTGGTCCCACCTATAA; *SP7* fw: CACAAAGAAGCCGTACTCTGT; *SP7* rev: GGGGCTGGATAAGCATCCC; *SPP1* fw: GAAGTTTCGCAGACCTGACAT; *SPP1* rev: GTATGCACCATTCAACTCCTCG.

Mouse primers:

*Alpl* fw: TTGTGCCAGAGAAAGAGAGAGA; *Alpl* rev: GTTTCAGGGCATTTTTCAAGGT; *Bmp2* fw: TCTTCCGGGAACAGATACAGG; *Bmp2* rev: TGGTGTCCAATAGTCTGGTCA; *Cbfa1* fw: AGAGTCAGATTACAGATCCCAGG; *Cbfa1* rev: AGGAGGGGTAAGACTGGTCATA; *Cdkn1a* fw: CCTGGTGATGTCCGACCTG; *Cdkn1a* rev: CCATGAGCGCATCGCAATC; *Gapdh* fw: AGGTCGGTGTGAACGGATTTG; *Gapdh* rev: TGTAGACCATGTAGTTGAGGTCA.

### Protein isolation and Western blotting

Total proteins were isolated by using ice-cold Pierce IP lysis buffer containing complete protease and phosphatase inhibitors cocktail (all from Fisher Scientific) and protein concentrations were determined by the Bradford assay (Bio-Rad Laboratories). Equal amounts of protein were boiled in Roti-Load1 Buffer (Carl Roth) at 100° C for 10 minutes and then separated on SDS-PAGE gels and transferred to PVDF membranes (Roche Applied Science). Membranes were incubated with primary antibodies: rabbit anti-phospho-p38 MAPK (Thr^180^/Tyr^182^) (1:1000, 9215, Cell Signaling), rabbit anti-p38 MAPK (1:1000, 9212, Cell Signaling), rabbit anti-phospho-SAPK/JNK (Thr^183^/Tyr^185^) (1:1000, 4668, Cell Signaling), rabbit anti-SAPK/JNK (1:1000, 9258, Cell Signaling), rabbit anti-phospho-p44/42 MAPK (Thr^202^/Tyr^204^) (1:1000, 4379, Cell Signaling), rabbit anti-p44/42 MAPK (1:1000, 4695, Cell Signaling), rabbit anti-phospho-DUSP1 (Ser^359^) (1:1000, 2857, Cell Signaling), rabbit anti-DUSP1 (1:1000, 35217, Cell Signaling) and rabbit anti-GAPDH (1:3000, 2118, Cell Signaling) at 4° C overnight and with secondary anti-rabbit HRP-conjugated antibody (1:1000, Cell Signaling) at room temperature for 1 hour. Membranes were stripped with Restore Plus Western blot stripping buffer (Fisher Scientific) at room temperature. Bands were detected with Clarity Western ECL substrate (Bio-Rad Laboratories) using the ChemiDoc MP imaging system (Bio-Rad Laboratories) and quantified using the ImageJ software. Data are shown as the ratio of phosphorylated to total protein to GAPDH, phosphorylated protein to GAPDH and of total protein to GAPDH, normalized to the control group [33, 59].

### Immunofluorescence staining and confocal microscopy

Cells were fixed in 4% PFA/PBS for 15 minutes, permeabilized in 0.3% TritonX-100/PBS for 10 minutes and blocked with 5% goat serum in 0.1% TritonX-100/PBS for 1 hour at room temperature. Cells were incubated with primary rabbit anti-RUNX2 antibody (1:100 in 0.1% TritonX-100/PBS, 12556, Cell Signaling) [54] at 4° C overnight and then with goat anti-rabbit Alexa488-conjugated antibody (1:500 in 0.1% TritonX-100/PBS, Invitrogen) for 2 hours at room temperature. Nuclei were stained with 0.5 μg/ml DAPI (Carl Roth) for 5 minutes at room temperature and slides were mounted with Prolong Diamond antifade reagent (Invitrogen). Images were acquired on a Nikon Ti-2 microscope (x60 oil immersion, NA 1.42) equipped with a Clarity Laser Free Confocal Unit (Aurox).

### Senescence-associated (SA)-β-galactosidase staining

HAoSMCs were stained for SA-β-Galactosidase by using the Senescence β-Galactosidase staining kit (9860, Cell Signaling).

### ALP activity

ALP activity was determined in cell lysates by using a colorimetric kit (Abcam) and protein concentration was determined by the Bradford assay (Bio-Rad Laboratories). Data are shown normalized to total protein concentration and to the control group [49].

### Calcification analysis

HAoSMCs were incubated with OsteoSense 680EX (1:250, NEV10020EX, Perkin Elmer) at 37° C overnight and images were acquired with the ChemiDoc MP imaging system (Bio-Rad Laboratories) [60, 61]. HAoSMCs and aortic tissues were decalcified in 0.6M HCl at 4° C and 37° C, respectively overnight and the calcium content in the supernatant was quantified by using the QuantiChrom Calcium assay kit (DICA-500, BioAssay Systems). Proteins were isolated by using 0.1M NaOH/0.1% SDS buffer and quantified by the Bradford assay (Bio-Rad Laboratories). Data are shown normalized to total protein concentration and to the control group.

### Statistics

Data are shown as scatter dot plots and arithmetic means ± SEM and *n* represents the number of independent experiments performed. Normalized data are shown as arbitrary units (a.u.). Normality was determined by Shapiro-Wilk test. For two groups, statistical testing was performed using unpaired T-test, Mann-Whitney-U-test or one-sample T-test. For multiple group comparison, statistical testing was performed by using one-way ANOVA with Tukey test (homoscedastic data) or Games-Howell test (heteroscedastic data) and Kruskal-Wallis test with Steel-Dwass test (non-normal data). *P*<0.05 was considered statistically significant.

## Supplementary Material

Supplementary Figures

Supplementary Table 1
