# Supplementary material for: Fisetin ameliorates vascular smooth muscle cell calcification via DUSP1-dependent p38 MAPK inhibition
Source: Aging (Albany NY). 2025 Apr 2;17(4):885–907. doi: 10.18632/aging.206233 (PMC12074812; doi:10.18632/aging.206233)
Supplement: Supplementary Figures [file aging-17-206233-s001.pdf]

SUPPLEMENTARY FIGURES

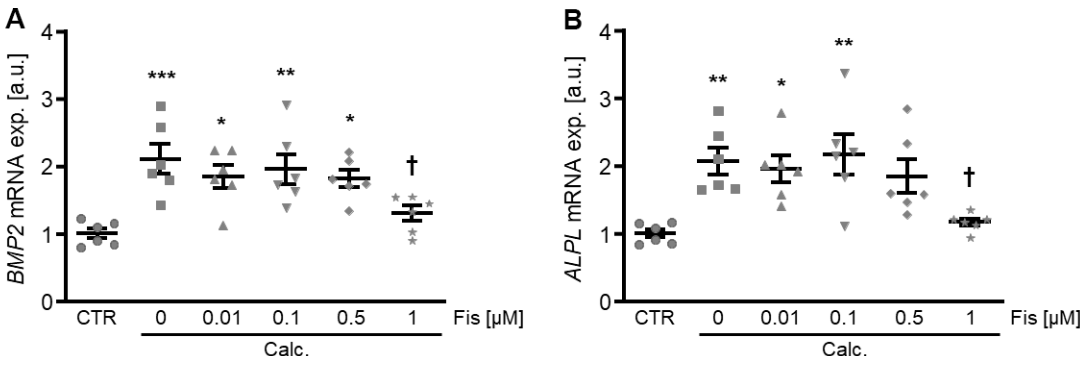

Supplementary Figure 1. Effects of low doses of fisetin on calcific marker expression in VSMCs during pro-calcific conditions. Relative mRNA expression (n=6) of *BMP2* (**A**) and *ALPL* (**B**) in HAoSMCs treated for 48h with control (CTR) or calcification medium (Calc.) without and with the indicated concentrations of fisetin (Fis, 0 - 1 µM). *(p<0.05), **(p<0.01), ***(p<0.001) significant vs. control group; †(p<0.05) significant vs. Calc.-treated group.

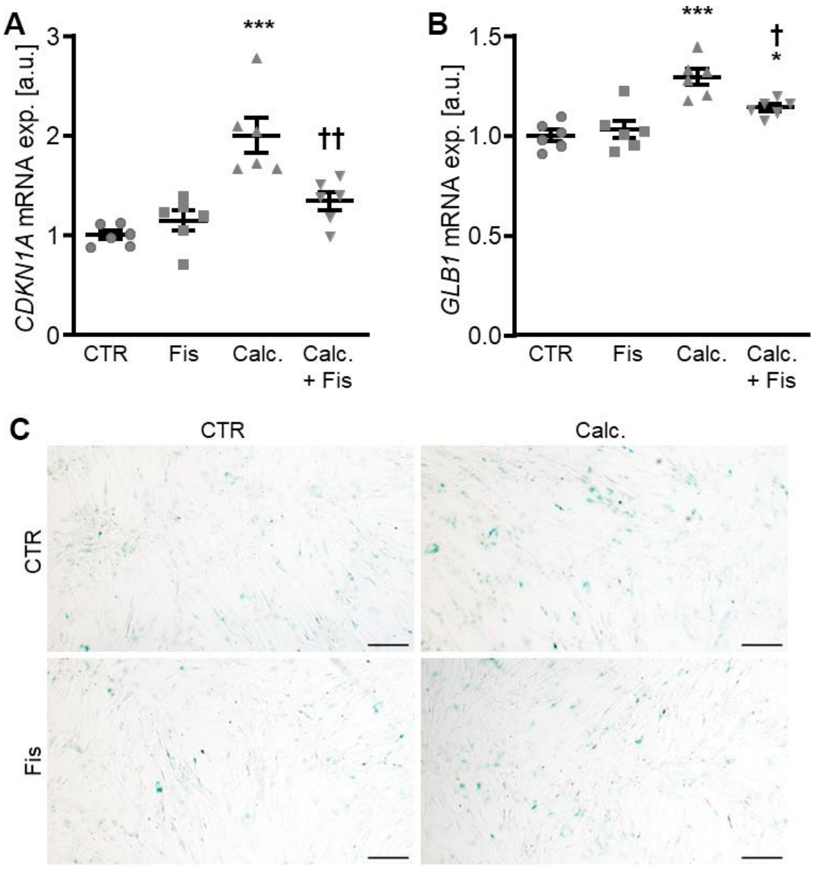

Supplementary Figure 2. Effects of fisetin on senescence markers in VSMCs during pro-calcific conditions. Relative mRNA expression (n=6) of *CDKN1A* (**A**) and *GLB1* (**B**) in HAoSMCs treated for 48h with control (CTR) or calcification medium (Calc.) without and with 1 µM fisetin (Fis). *(p<0.05), ***(p<0.001) significant vs. control group; †(p<0.05), ††(p<0.01) significant vs. Calc.-treated group. (**C**) Senescence-associated (SA)-β-galactosidase staining in HAoSMCs treated for 5d with control (CTR) or calcification medium (Calc.) without and with 1 µM fisetin (Fis). SA-β-galactosidase positive cells: blue-green; scale bar: 250 µm.

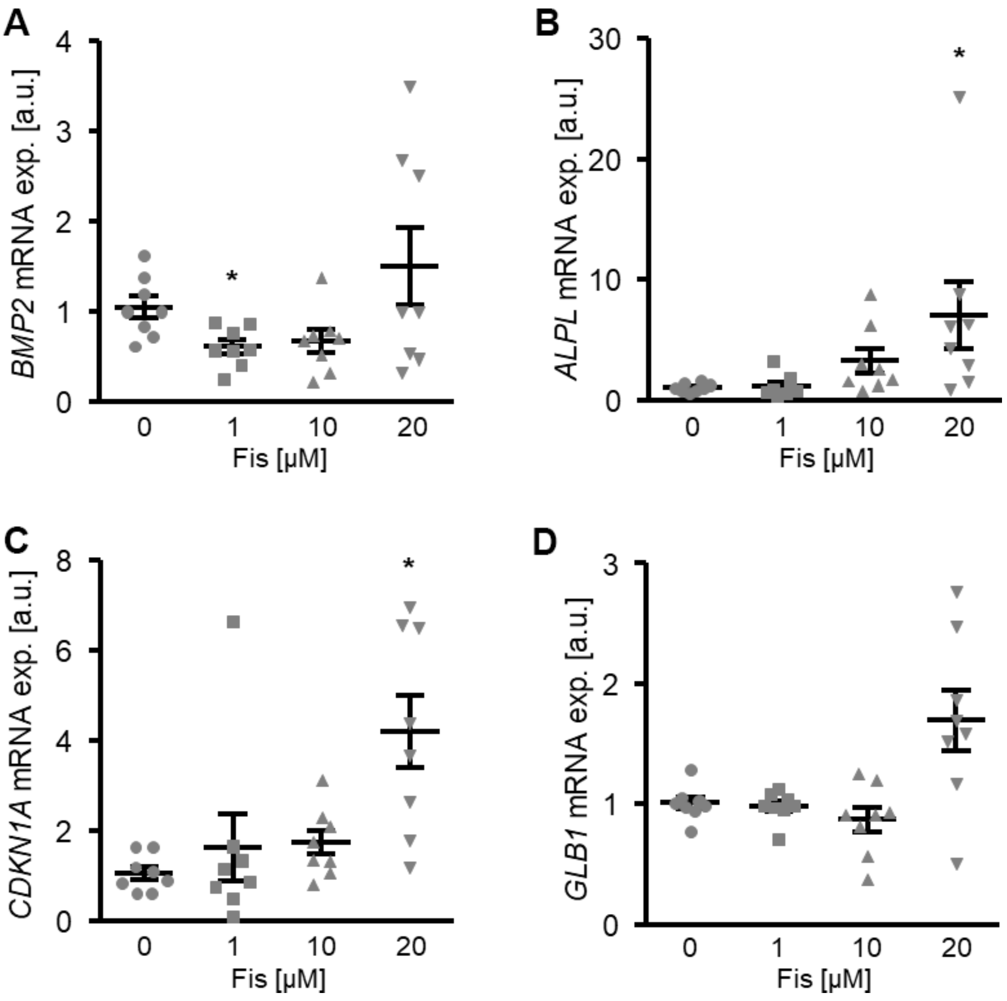

Supplementary Figure 3. Effects of fisetin on calcific and expression in VSMCs. Relative mRNA expression (n=8) of *BMP2* (**A**), *ALPL* (**B**), *CDKN1A* (**C**) and *GLB1* (**D**) in HAoSMCs treated for 48h without and with the indicated concentrations of fisetin (Fis, 0 - 20 µM). *(p<0.05) significant vs. control group.

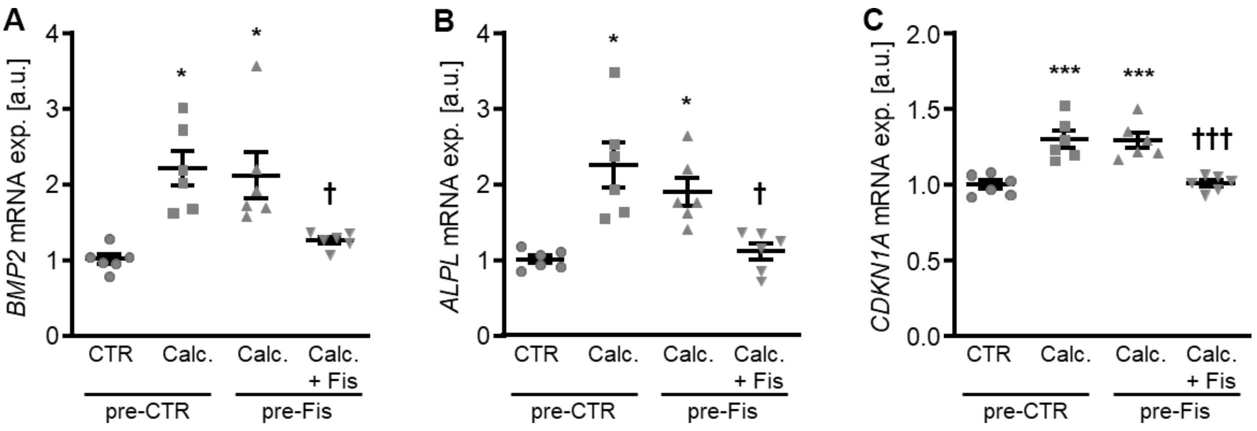

Supplementary Figure 4. Effects of fisetin preincubation on calcific marker expression in VSMCs during pro-calcific conditions. Relative mRNA expression (n=6) of *BMP2* (**A**), *ALPL* (**B**) and *CDKN1A* (**C**) in HAoSMCs pre-treated for 48h with control (CTR) or 1 µM fisetin (Fis) and treated for additional 48h with control (CTR) or calcification medium (Calc.) without and with 1 µM fisetin (Fis). *(p<0.05), ***(p<0.001) significant vs. control group; †(p<0.05), †††(p<0.001) significant vs. CTR-pre-treated and Calc.-treated group.

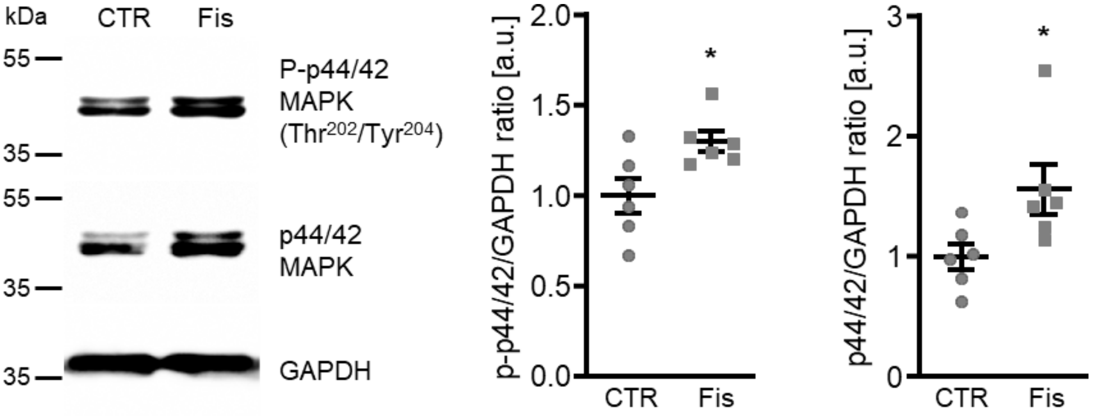

Supplementary Figure 5. Effects of fisetin on ERK1/2 MAPK regulation in VSMCs. Representative Western blots and normalized phospho-p44/42 and total p44/42 MAPK protein abundance (n=6) in HAoSMCs treated for 30min with control (CTR) or 1 µM fisetin (Fis). *($p<0.05$) significant vs. control group.

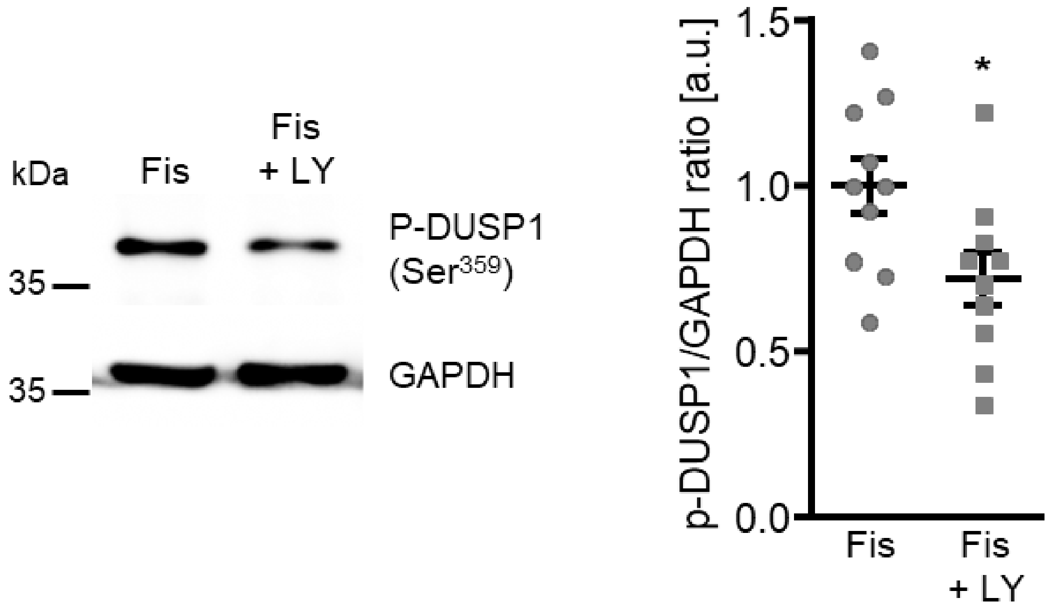

Supplementary Figure 6. Effects of ERK1/2 MAPK inhibition on DUSP1 regulation in VSMCs. Representative Western blots and normalized phospho-DUSP1 protein abundance (n=10) in HAoSMCs treated for 30min with 1 µM fisetin (Fis) without and with 1 µM p44/42 MAPK inhibitor LY3214996 (LY). *($p<0.05$) significant vs. Fis-treated group.

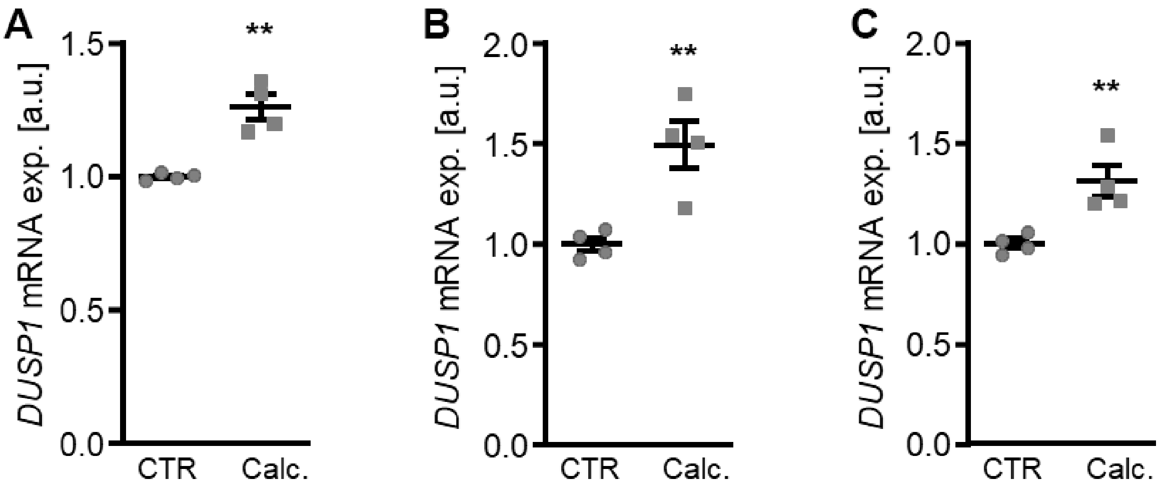

Supplementary Figure 7. Dual-specificity phosphatase 1 expression in VSMCs during pro-calcific conditions. Relative mRNA expression (n=4) of *DUSP1* in HAoSMCs treated for 2h (**A**), 24h (**B**) and 48h (**C**), respectively with control (CTR) or calcification medium (Calc.). **(p<0.01) significant vs. control group.

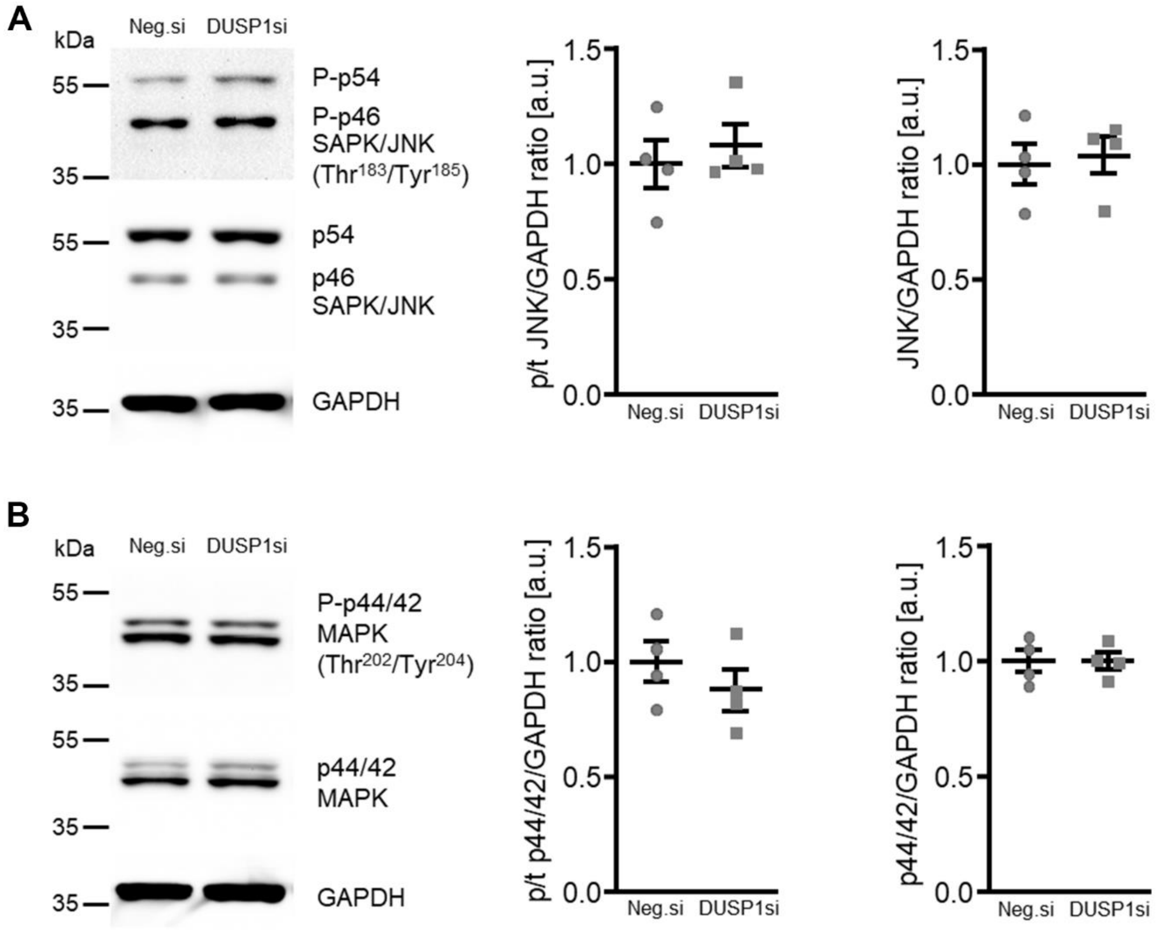

Supplementary Figure 8. Effects of dual-specificity phosphatase 1 knockdown on SAPK/JNK and p44/42 MAPK phosphorylation in VSMCs. Representative Western blots and normalized phospho-SAPK/JNK and total SAPK/JNK protein abundance (n=4, **A**) as well as phospho-p44/42 MAPK and total p44/42 MAPK protein abundance (n=4, **B**) in HAoSMCs transfected for 24h with negative control (Neg.si) or DUSP1 (DUSP1si) siRNA.

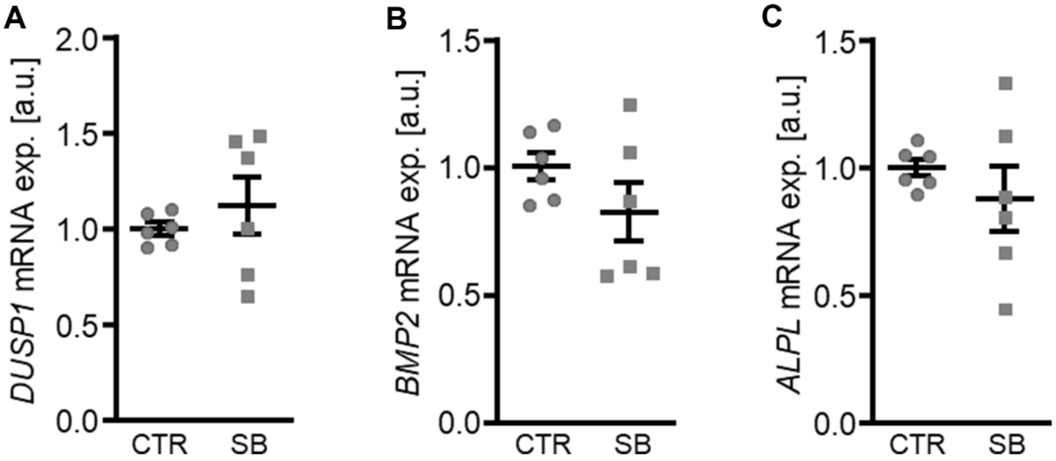

Supplementary Figure 9. Effects of p38 MAPK inhibitor SB203580 on dual-specificity phosphatase 1 and calcific marker expression in VSMCs. Relative mRNA expression (n=6) of *DUSP1* (**A**), *BMP2* (**B**) and *ALPL* (**C**) in HAoSMCs treated for 72h without and with 10 µM p38 MAPK inhibitor SB203580 (SB).

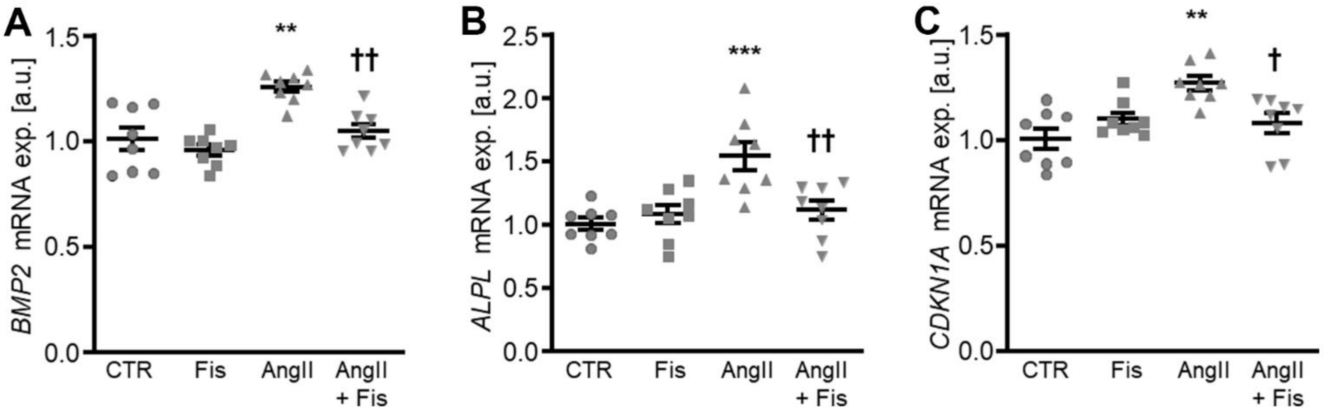

Supplementary Figure 10. Effects of fisetin on angiotensin II-induced calcific marker expression in VSMCs. Relative mRNA expression (n=8) of *BMP2* (**A**), *ALPL* (**B**) and *CDKN1A* (**C**) in HAoSMCs treated for 24h with control (CTR) or 100 nM angiotensin II (AngII) without and with 1 µM fisetin (Fis). **($p<0.01$), ***($p<0.001$) significant vs. control group; †($p<0.05$), ††($p<0.01$) significant vs. AngII-treated group.

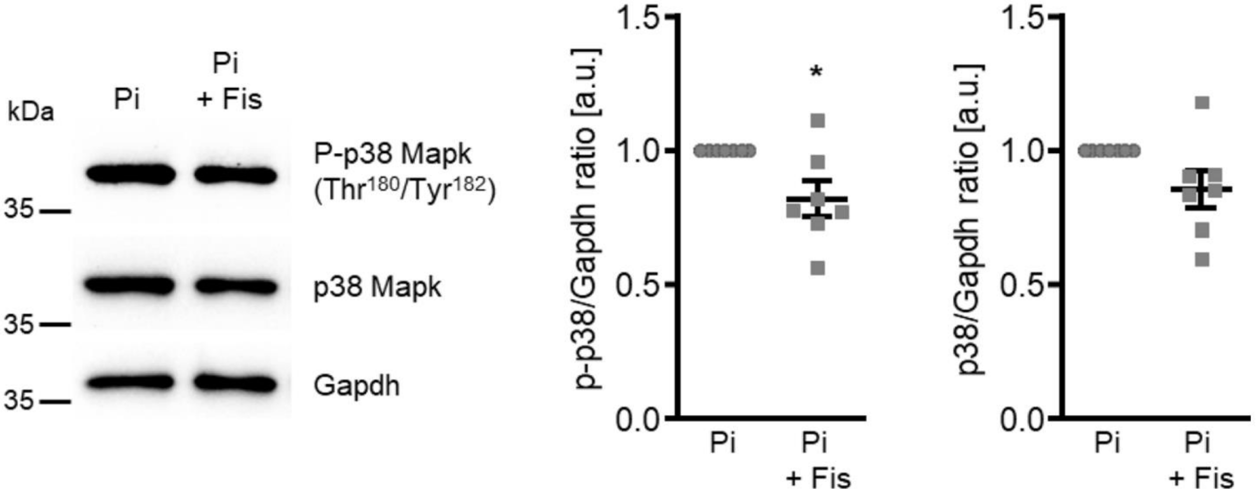

Supplementary Figure 11. Effects of fisetin on p38 Mapk activation *ex vivo* in calcifying mouse aortic explants. Representative Western blots and normalized phospho-p38 and total p38 Mapk protein abundance (n=7) in mouse aortic explants cultured for 1h in medium supplemented with 1.6 mM phosphate (Pi) without and with 1 µM fisetin (Fis). *(p<0.05) significant vs. Pi-treated group.
