# Supplementary material for: Fisetin ameliorates vascular smooth muscle cell calcification via DUSP1-dependent p38 MAPK inhibition
Source: Aging (Albany NY). 2025 Apr 2;17(4):885–907. doi: 10.18632/aging.206233 (PMC12074812; doi:10.18632/aging.206233)
Supplement: Supplementary Table 1 [file aging-17-206233-s002.pdf]

SUPPLEMENTARY TABLE

Supplementary Table 1. Effects of fisetin in mice during cholecalciferol overload.

	CTR	Fis	vD	vD + Fis	
Calcium [mg/dl]	10.18±0.46	10.47±0.38	24.36±1.06**	17.89±0.67**,†	n=6-9
Phosphate [mg/dl]	9.42±0.42	8.5±0.32	5.72±0.15***	8.47±0.64†	n=6-9
Cystatin C [ng/ml]	685.95±83.69	867.78±76.40	1125.80±99.28**	1059.03±70.54*	n=6-9
Fetuin A [µg/ml]	158.94±11.88	161.02±4.37	112.55±6.75**	159.80±9.26††	n=6-9

Serum calcium, phosphate, Cystatin C and Fetuin A levels in mice receiving vehicle (CTR) or high-dosed cholecalciferol (vD) without and with fisetin (Fis). *(p<0.05), **(p<0.01), ***(p<0.001) significant vs. control group; †(p<0.05), ††(p<0.01) significant vs. vD-treated group.
